# HOST PLANT UTILIZATION, HOST RANGE OSCILLATIONS AND DIVERSIFICATION IN NYMPHALID BUTTERFLIES: A PHYLOGENETIC INVESTIGATION

**DOI:** 10.1111/evo.12227

**Published:** 2013-08-29

**Authors:** Sören Nylin, Jessica Slove, Niklas Janz

**Affiliations:** 1 Department of Zoology, Stockholm UniversityStockholm, 10691, Sweden

**Keywords:** Phenotypic plasticity, phylogenetics, plant–insect interaction, speciation

## Abstract

It has been suggested that phenotypic plasticity is a major factor in the diversification of life, and that variation in host range in phytophagous insects is a good model for investigating this claim. We explore the use of angiosperm plants as hosts for nymphalid butterflies, and in particular the evidence for past oscillations in host range and how they are linked to host shifts and to diversification. At the level of orders of plants, a relatively simple pattern of host use and host shifts emerges, despite the 100 million years of history of the family Nymphalidae. We review the evidence that these host shifts and the accompanying diversifications were associated with transient polyphagous stages, as suggested by the “oscillation hypothesis.” In addition, we investigate all currently polyphagous nymphalid species and demonstrate that the state of polyphagy is rare, has a weak phylogenetic signal, and a very apical distribution in the phylogeny; we argue that these are signs of its transient nature. We contrast our results with data from the bark beetles *Dendroctonus*, in which a more specialized host use is instead the apical state. We conclude that plasticity in host use is likely to have contributed to diversification in nymphalid butterflies.

The view of the role of phenotypic plasticity in evolution has changed dramatically over the last two decades, due in large part to the influence of West-Eberhard's proposal that plasticity may fuel diversification (West-Eberhard 1989, 2003; Pfennig et al. 2010). Rather than evolution waiting on mutations for the creation of new genetic variation, it may be the case that organisms initially cope with new situations by making use of existing phenotypic plasticity in trait expression (this view is related to the concept of “ecological fitting”; Agosta et al. 2010). If organisms are thus able to persist in a new environment, selection may ultimately be able to favor novel genetic variants that confer superior performance under those conditions (West-Eberhard 2005a, b).

With this view of evolution, genes are “followers” rather than “leaders” (West-Eberhard 2003; but see also Schwander and Leimar 2011 for a more nuanced view). If plasticity can allow organisms to persist in new environments or use novel resources, and facilitate adaptation to those conditions, plasticity may also contribute to speciation and diversification. Note that here we follow West-Eberhard (2003) in our use of the term “phenotypic plasticity” in that we do not make a strong distinction between the many ways it can be applied. In particular, we assume that “environmental plasticity” (when a range of traits are conditionally expressed in response to the environment) and “evolutionary plasticity” (which could be defined as a propensity for new traits to evolve and for ancestral phenotypes to be lost and appear again repeatedly in a clade) are fundamentally linked through West-Eberhard's concept of “developmental plasticity.” That is, both phenomena suggest the presence of genetic and developmental “machinery” capable of producing a range of phenotypic traits, even though some traits may not be expressed for long periods of time (West-Eberhard 2003, and see also West-Eberhard 2005a, b2005b, 2008).

It has been suggested that plasticity in host use by phytophagous insects may be a particularly good test case for investigating the importance of plasticity in speciation and diversification (Nylin and Janz 2009; Dennis et al. 2011). Indeed, there is some evidence that plastic host utilization contributes to diversification among nymphalid butterflies, in that taxa with more diverse host use have more species than sister taxa feeding on a more restricted set of hosts (Janz et al. 2006; Nylin and Wahlberg 2008). This evidence is however indirect in the sense that it is based on diversity of host use in higher taxa, not plasticity within species. Using such indirect evidence may be necessary, because host range is likely to be evolutionarily dynamic and thus not prone to phylogenetic reconstruction with much certainty (Stireman 2005). However, the two forms of plasticity may be connected and, furthermore, it can be argued that diverse host use in, for example, a genus can only evolve via plastic evolutionary stages where both old and new hosts are used by the same species at some point in time, probably most often in combination with a more extended set of hosts (Janz et al. 2006; Nylin and Wahlberg 2008).

It would however be of great interest to investigate host range at the species level and its role in evolution, as far as this is at all possible. This is because current theory on exactly how host plasticity could contribute to diversification is based on oscillations in host range over evolutionary time, with ancestral specialists giving rise to plastic and widespread generalists that in turn would tend to adapt to local conditions and diversify (Janz and Nylin 2008). Thus, we have assembled the available evidence on host range in Nymphalidae at the species level, and will here focus on polyphagy and evidence of its transient nature, as suggested by the “oscillation hypothesis.”

First, however, we will “set the scene” for these evolutionary dynamics, by mapping the data on host orders for nymphalid butterflies onto the phylogenies for the family that have only recently become available, thus reconstructing the main patterns of host use and the major transitions among host orders over evolutionary time. This will also provide the opportunity to compare the form and strength of the phylogenetic signals of host use (i.e., precisely which plant orders are used) and niche width (here the number of plant orders used), respectively.

## Materials and Methods

### SOURCES OF HOST DATA

We scrutinized the literature on local butterfly faunas for host plant records. The main sources were: Ebert (1993), Tolman and Lewington (1997), Tuzov (2000) (for Europe); Corbet and Pendlebury (1992), Igarashi and Fukuda (1997), Bascombe et al. (1999), Igarashi and Fukuda (2000), Tuzov (2000) (for Asia); Gibbs (1980), Parsons (1991), Braby (2000), Tennent (2002) (for the Australian region and the Pacifics); Migdoll (1987), Larsen (1991), Tennent (1996), Larsen (2005) (for Africa); Scott (1986), Layberry et al. (1998) (for North America); and DeVries (1987), Smith et al. (1994) (for Central and South America, West Indies). Furthermore, we investigated all records reported on the website of Savela (2011) against the primary literature and made use of literature on specific butterfly taxa including host plant records: Ackery and Vane-Wright (1984, for Danaini), Penz et al. (2000, for Brassolini), Wahlberg (2000, for Melitaeini), and Willmott and Freitas (2006, for Ithomiinae).

We were primarily interested in assembling records at the level of host orders, and thus a record was deemed more reliable if more than one taxon in the same plant order have been reported as hosts, but also if there were independent records of the same host taxa or if detailed information was available on the nature of the actual observations behind the record. If it was difficult to judge the reliability of a host order record because it came from a single source without such information, it was included in the database (Appendix S1) but set in parentheses and disregarded for the analyses reported here.

### PLANT PHYLOGENY AND TAXONOMY

We used the Angiosperm Phylogeny Website (Stevens 2001) to assign host records to currently recognized plant orders. A few plant families used as hosts are still unplaced, and for the purposes of the analyses they were treated as independent taxa at the level of orders. For simplicity, we made no additional use of information on the phylogenetic relationships among plants beyond placing families in higher taxa. Orders (or any level in the taxonomical hierarchy) are arbitrarily defined and not directly comparable in terms of being of equal age, but for the purposes of this study this is not likely to be an important limitation. Information on plant relationships and phylogenetic distances between taxa is however constantly being improved and future studies on insect–plant relationships should attempt to take this information explicitly into account. Furthermore, phytophagous insects do not interact with plant taxa per se, but rather with plant chemistry, physiology, and morphology, and more complete databases on such properties with information that could be readily compared across plant taxa would be highly desirable. At present, plant taxonomy is however still a useful proxy.

### BUTTERFLY PHYLOGENY AND TAXONOMY

The taxonomy of Nymphalidae has changed drastically over the past few years, due to a much improved phylogenetic understanding (especially Wahlberg et al. 2003, 2005, 2009; Brower et al. 2006, 2010; Pena et al. 2006; Simonsen et al. 2006; Willmott and Freitas 2006; Silva-Brandao et al. 2008). We closely followed the species lists and delimitations of higher butterfly taxa suggested by the Tree of Life Website (Maddison and Schulz 2007), as this section of the website is well curated and provides a “point of stability” in the taxonomy. We used the program Mesquite (Maddison and Maddison 2011) to create the phylogeny, starting with a list of all nymphalid genera recognized in Tree of Life, deleting taxa without host plant information, presenting the remaining taxa as a nonresolved bush, and then manually resolving the phylogeny by moving branches.

For the Nymphalidae phylogeny itself we also incorporated well-supported changes suggested more recently (in particular in Wahlberg et al. 2009), but not yet reflected in Tree of Life. Additional sources were: Kodandaramaiah et al. (2010a, b2010b, Mycalesina, Coenonymphina); Ohshima et al. (2010, Apaturinae); Pena et al. (2010, 2011, Euptychiina, Satyrini); Price et al. (2011, Dirini); Ortiz-Acevedo and Wilmott (2013, Preponini), and Penz et al. (2013, Brassolini). It should be noted that to make full use of this information, we resolved a few sections of the phylogeny while leaving taxa not included in the studies listed above as unresolved at the base of the respective clades, rather than collapsing the entire clades as would have been the strictly correct procedure. However, we also tested the effects of using a more conservative phylogeny in accordance with the Tree of Life site (as of 2012). The results were very similar and are not shown here.

Furthermore, we obtained some information on branch lengths in the complete phylogeny by incorporating ages of all dated nodes in Wahlberg et al. (2009), which corresponded to nodes in our phylogeny, using the tool “Node age constraints” in the program Mesquite (Maddison and Maddison 2011). Ages of nodes were added as “fixed,” but branch lengths were then transformed using the option Enforce Minimum Node Age Constraints. Using this method it was possible to obtain branch length estimates also for sections of the phylogeny containing taxa with host plant information not included in Wahlberg et al. (2009) or where more recently proposed topologies are inconsistent with this study. However, to avoid zero-length branches we had to arbitrarily give all splits between two most closely related terminal taxa for which age information was lacking an arbitrary minimum age of 10 Mya (most dated splits of this kind in the phylogeny are in the range 12–20 Mya).

Our final database of the Nymphalidae host records (Appendix S1) contains 551 butterfly taxa. In the large majority of cases these taxa are at the genus level, but a few are species that are currently unplaced. In the phylogenetic analyses, moreover, the diverse satyrine tribe Pronophilini, with 66 neotropical genera only recorded to feed on Poales, was for simplicity treated as a single taxon (underestimating slightly the very conserved nature of use of Poales in the Satyrinae). All available host plant records were investigated at the species level, and the 551 taxa in the database thus represents about 6000 species in the family (Maddison and Schulz 2007) to the extent that there are host plant records at the species level.

**Figure 1:**
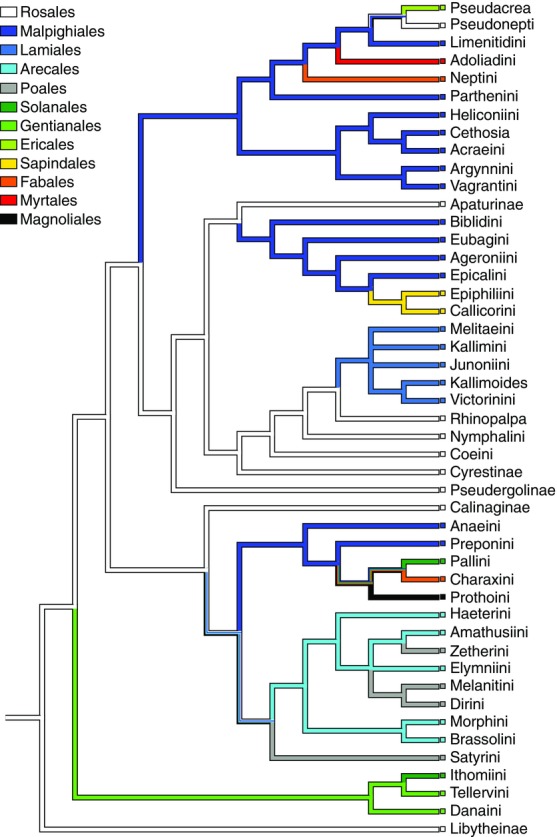
Character optimization of main host order utilization (transitions unordered) on a simplified phylogeny of nymphalid butterflies.

**Figure 2:**
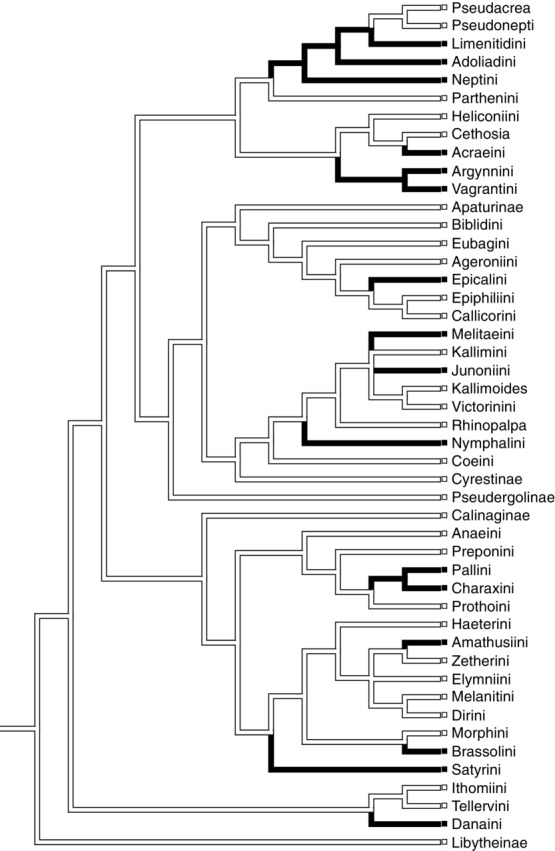
Character optimization of polyphagy (use of at least three orders, transitions unordered) on a simplified phylogeny of nymphalid butterflies. Lineages containing polyphagous species in black. Note that a butterfly taxon is coded as polyphagous if it contains a single polyphagous species.

For purposes of overview and illustration, we also made use of a simplified phylogeny of Nymphalidae at the level of tribes (or subfamilies for some smaller taxa; Figs. 1, 2).

### RECONSTRUCTION OF PAST HOST USE

We first used parsimony optimization (as implemented in Mesquite; Maddison and Maddison 2011) to reconstruct host plant use by tracing the “main” host order used in each butterfly taxon (coded as a single multistate character) onto the simplified phylogeny shown in Figure 1. Assigning the main host orders (Table 1) was in most cases straightforward, but in some cases a more arbitrary and qualitative decision. The main criterion aiding the choice was host orders used across the majority of genera in tribal-level butterfly taxa, in particular if some genera are specialized on the plant order. For the more arbitrary choices we also took some impression from comments in regional butterfly faunas concerning the importance of particular plant taxa as hosts across butterfly species.

**Table 1:** Main host orders and polyphagy (feeding on three orders or more) mainly at the level of tribes of nymphalid butterflies. Main hosts indicated by asterisk are somewhat arbitrary choices (see main text for more details). Families mentioned in host comments are main host families, not exhaustive list. Species mentioned in polyphagy comments are dubiously polyphagous, in taxa lacking polyphagy

Taxon	Main host	Comment	Polyphagy	No. of species	No. of genera	Total no. of genera	Comment
Libytheinae	Rosales	Cannabaceae	No			2	*Libythea geoffroy*?
Parthenini	Malpighiales	Several families	No			3	*Parthenos sylvia*?
Neptini	Fabales^*^	Malpighiales etc. also important	Yes	10	2	7	
*Pseudoneptis*	Rosales	Moraceae	No			1	
Adoliadini	Myrtales^*^	Malpighiales etc. also important	Yes	4	4	23	
Limenitidini	Malpighiales^*^	Many orders	Yes	3	2	14	
*Cethosia*	Malpighiales	Passifloraceae	No			1	
Heliconiini	Malpighiales	Passifloraceae	No			9	
Acraeini	Malpighiales^*^	Many orders	Yes	8	2	2	
Vagrantini	Malpighiales	Salicaceae	Yes	1	1	10	
Argynnini	Malpighiales	Violaceae	Yes	5	2	7	
Apaturinae	Rosales	Cannabaceae	No			19	
Biblidini	Malpighiales	Euphorbiaceae	No			10	
Epicalini	Malpighiales	Euphorbiaceae	Yes	1	1	7	
Ageroniini	Malpighiales	Euphorbiaceae	No			4	
Epiphilini	Sapindales	Sapindaceae	No			8	
Eubagini	Malpighiales	Euphorbiaceae	No			1	
Callicorini	Sapindales	Sapindaceae	No			9	
Cyrestinae	Rosales	Moraceae	No			3	
Coeini	Rosales	Urticaceae	No			2	
Junoniini	Lamiales	Acanthaceae, Lamiaceae	Yes	4	2	6	
Melitaeini	Lamiales	Acanthaceae, Plantaginaceae	Yes	4	1	24	
Kallimini	Lamiales	Acanthaceae	No			4	*Doleschallia tongana*?
*Kallimoides*	Lamiales	Acanthaceae	No			1	
Victorinini	Lamiales	Acanthaceae	No			4	
*Rhinopalpa*	Rosales	Urticaceae	No			1	
Nymphalini	Rosales	Urticaceae	Yes	7	3	14	
Pseudergolinae	Rosales	Urticaceae	No			4	
Charaxini	Fabales^*^	Many orders	Yes	21	2	3	
Pallini	Solanales^*^	Convolvulaceae	Yes	1	1	1	
Prothoini	Magnoliales	Annonaceae	No			2	
Anaeini	Malpighiales	Euphorbiaceae	No			10	
Preponini	Malpighiales^*^	Several orders	No			4	
Elymnini	Arecales^*^	Several monocot orders	No			4	
Zetherini	Poales^*^	Several monocot orders	No			6	
Amathusiini	Arecales^*^	Several monocot orders	Yes	3	2	12	
Brassolini	Arecales^*^	Several monocot orders	Yes	3	2	18	
Morphini	Arecales^*^	Several orders	No			3	
Melanitini	Poales	Poaceae	No			6	
Dirini	Poales	Poaceae	No			6	
Haeterini	Arecales^*^	+ Zingiberales	No			5	
Satyrini	Poales	Poaceae	Yes	1	1	206	
Calinaginae	Rosales	Moraceae	No			1	
Ithomiini	Solanales	Solanaceae	No			45	
Danaini	Gentianales	Apocynaceae, Asclepiadaceae	Yes	3	2	12	
Tellervini	Gentianales	Apocynaceae	No			1	
Total				79	30	545	

For the actual reconstructions, however, we used a phylogeny of the 418 (primarily genus level) butterfly taxa in our database for which we had found host plant records, first resolving the phylogeny in accordance with Tree of Life (Maddison and Schulz 2007) and then modifying the tree to reflect the newer literature listed earlier. Parsimony optimizations were performed for each host order separately, coded as independent 0/1 characters. Gains and losses of host orders were weighed either equally, or with gains weighed as being more unlikely than losses (“costly gains”; weight 2 vs. 1) or much more unlikely (“very costly gains”; 5 vs. 1) using a step-matrix to implement the costs of gains versus losses. Utilization of a plant taxon as a butterfly host necessitates finding, recognition, and acceptance by egg-laying females as well as acceptance and successful feeding by their offspring, and it seems reasonable to assume that in butterfly evolution it is easier to lose an association with a particular host than to first evolve such a complex trait.

### ANALYSES OF POLYPHAGY

Most of the taxa in our butterfly database (in most cases genera) completely lack species that have been recorded to feed on more than one host plant order (note that the orders listed for each higher taxon is the complete list of orders reliably recorded from this taxon, and can be from a number of specialists rather than from a species feeding on several orders). We defined a “polyphagous” species as one having larvae that feed on at least two orders (character “2 orders”) or at least three orders (character “3 orders”), and the database (Appendix S1) lists all and only such species (along with some where the host records do not currently permit us to decide whether they should be deemed polyphagous according to these criteria or not).

To quantify the phylogenetic distribution of these states of polyphagy, we used both methods based on parsimony and on maximum likelihood (Pagel 1999), contrasting the results with those obtained from analyzing the distribution of host character states (the use of each order across the phylogeny) with the same methods. We also applied the same analyses to a data set from the bark beetle genus *Dendroctonus* (Kelley and Farrell 1998), which is of interest because it has been subject to discussion regarding whether it shows mostly transitions from a generalized to a more specialized host use or rather the reverse (Kelley and Farrell 1998; Nosil 2002; Stireman 2005).

First, we investigated the *phylogenetic signal* in these characters, that is whether closely related species are more likely to share a character state than expected from chance. This was done in two ways: performing a randomization test (parsimony method) and calculating Pagel's λ (likelihood method). The randomization test was performed in Mesquite (Maddison and Maddison 2011), comparing the number of steps in each character for the given tree with 999 random trees created by reshuffling of terminal taxa. Lambda was estimated for each character using the *fitDiscrete* function in the Geiger package (Harmon et al. 2008) in R 2.13 (R Development Core Team 2008). Because branch lengths are not available for the complete phylogeny, we tested the effects of either rendering the tree ultrametric by transforming branch lengths using the “arbitrarily ultrametricize” function in Mesquite, or enforcing minimum node age constraints as described earlier. The results were qualitatively very similar and only results from the enforced minimum node ages are shown here.

Second, as a complementary analysis, we investigated the *degree of homoplasy* and *apicality* in the characters, that is whether the same character state tends to evolve only once or rarely (more likely to result in the same state being shared by relatives and being optimized as going “deep” into the phylogeny), or repeatedly and convergently (more likely to result in an apical distribution of the state in the phylogeny). This was done in two ways: calculating the *retention index* (parsimony method) and the *delta estimate* (likelihood method). The retention index (Farris 1989) was calculated for each character as well as for the complete character matrix in Mesquite. Delta was estimated in the same way as Pagel's λ. Delta describes if change mostly occurs in the tips or deep in the tree (where values below 1 represents change mainly occurring toward the base of the tree and above 1 change tends to occur more at the tips of the tree).

Third, we compared the frequencies of reconstructed transitions from monophagy to polyphagy or vice versa (using the “Summarize state changes in tree” option in Mesquite), reasoning that a higher frequency of transitions to polyphagy is indicative of an apical distribution of this character state.

### CASE STUDIES

We provide a closer view of the taxa with most widespread polyphagy and containing the most polyphagous nymphalid species, to illustrate more clearly the patterns revealed by the wider analyses and to scrutinize the available data on whether plasticity at the species level can be shown to aid diversification. These case studies are *Nymphalis*/*Polygonia*, *Vanessa*, *Hypolimnas*, and *Charaxes*.

**Figure 3:**
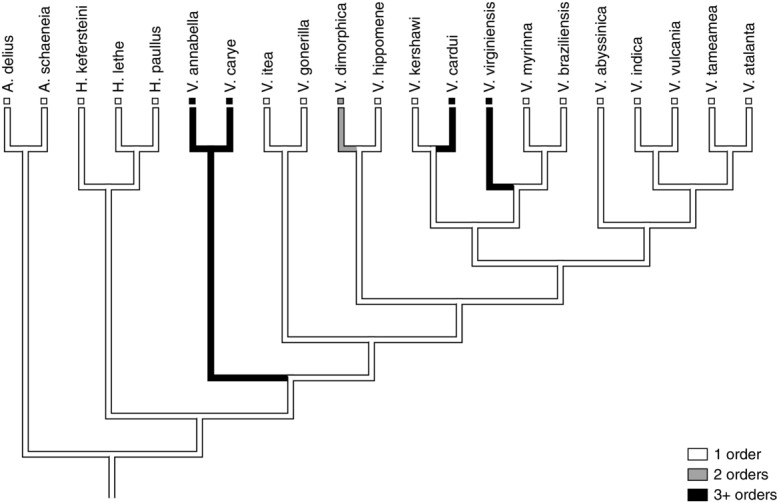
Character optimization of polyphagy on a phylogeny of *Vanessa* butterflies and the related genera *Hypanartia* and *Antanartia*, based on Wahlberg and Rubinoff (2011). Only species with host plant data included. Transitions unordered. Gray = two host orders; black = three or more host orders.

**Figure 4:**
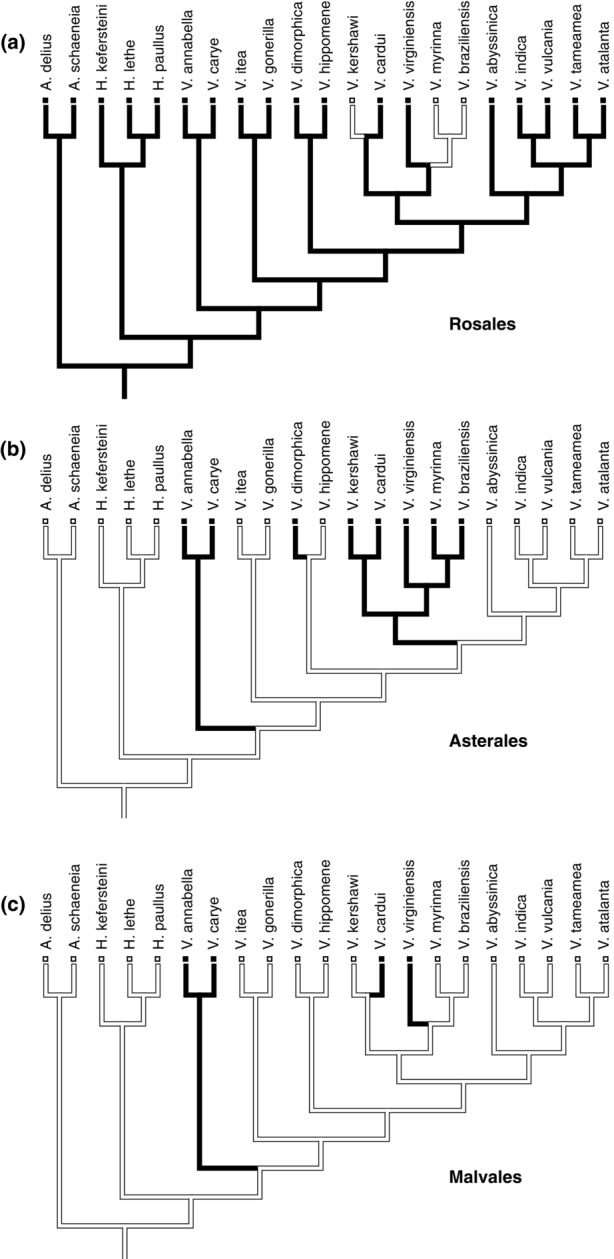
Character optimization of host use on a phylogeny of *Vanessa* butterflies and related genera, based on Wahlberg and Rubinoff (2011). Only species with host plant data included. Transitions unordered. Use of (a) Rosales; (b) Asterales; and (c) Malvales.

For the *Vanessa* group a phylogenetic hypothesis has recently become available (Wahlberg and Rubinoff 2011), and we make use of this phylogeny to illustrate host use and diversification at the species level, complementing our earlier studies on *Nymphalis/Polygonia*. Only species with host plant data were included, and note that the position of the genus *Antanartia* is still uncertain. It may be the sister to *Vanessa* + *Hypanartia* as in Figures 3 and 4, or the basal taxon in a clade also containing *Aglais*, *Nymphalis*, *Polygonia*, and relatives, as in Wahlberg and Rubinoff (2011). In any case, the taxon supports the ancestral state of specialization on Rosales, also shared with the even more basal genera in the tribe Nymphalini (*Mynes*, *Symbrenthia*, *Araschnia*; Janz and Nylin 2001; Nylin and Wahlberg 2008). Host plant data were taken from the sources listed earlier. A parsimony reconstruction was performed in Mesquite for the character “polyphagy,” treated as an unordered multistate character with the states: (0) one host order used; (1) two orders used; (2) three or more orders used. Use of the three main host orders for the genus, that is Rosales (in this case mainly the family Urticaceae), Asterales, and Malvales was reconstructed using parsimony and treating use of each order as a separate unordered binary character.

## Results

### GENERAL PATTERNS OF HOST USE IN THE NYMPHALIDAE

#### Host taxa

There are 65 angiosperm plant orders currently recognized, plus 10 as yet unplaced taxa (Stevens 2001). Out of these, there are reliable records of 33 orders being used as hosts by Nymphalidae, and in addition the family Boraginaceae from the unplaced taxa, and the gymnosperm order Selaginellales (used by a few satyrines; Murray and Prowell 2005). Despite this diversity of host use, however, only a handful of orders are “important” host orders; that is characteristic of higher clades (Table 1 and Fig. 1) and also fed upon by many genera (Table 2). These are: Rosales, Malpighiales, Gentianales, Solanales, Arecales, Poales, and Lamiales.

**Table 2:** Phylogenetic signal in polyphagy and host order utilization in nymphalid butterflies, according to parsimony (randomization test) and ML. The “no. of bars” (in the histogram resulting from the randomization test) is an indication of the variation in number of steps over 1000 random trees. When no. of steps in characters fall below percentile boundaries they are significant at *P* < 0.05 (marked in bold). ML lambda estimates significantly different from 0 with a *P*-value of <0.05 are marked in bold

		Parsimony			ML	
Character	No. of butterfly taxa	No. of bars	Steps in char.	Percentile boundary (0.05)	Lambda estimate	*P*-value
Two orders	71	16	**51**	61	0.77	**0.000**
Three orders	30	5	27	26	0.27	0.580
Arecales	24	6	**13**	21	0.78	**0.000**
Asparagales	4	2	3	3	0.87	**0.023**
Asterales	12	3	11	10	0.41	0.320
Boraginaceae	3	2	3	2	0.00	1.000
Brassicales	2	1	2	2	0.00	0.990
Caryophyllales	9	4	8	7	0.56	0.160
Celastrales	4	2	4	3	0.00	0.990
Commelinales	2	2	1	1	1.00	**0.002**
Cucurbitales	2	1	2	2	0.00	0.990
Dipsacales	6	2	6	5	0.00	0.990
Ericales	16	4	15	14	0.31	0.550
Fabales	12	4	**9**	10	0.92	**0.000**
Fagales	13	3	12	11	0.05	0.960
Gentianales	15	4	**5**	13	1.00	**0.000**
Lamiales	41	8	**19**	36	0.67	**0.000**
Laurales	16	3	15	14	0.60	0.260
Liliales	3	2	2	2	0.91	**0.013**
Magnoliales	3	2	3	2	1.00	0.400
Malphigiales	78	16	**26**	66	0.86	**0.000**
Malvales	13	4	11	11	0.79	0.058
Myrtales	14	4	13	12	0.32	0.640
Oxalidales	3	2	3	2	0.00	0.990
Pandanales	4	2	3	3	0.79	**0.031**
Piperales	4	2	4	3	0.00	0.990
Poales	109	24	**15**	87	1.00	**0.000**
Ranunculales	4	2	4	3	0.85	0.720
Rosales	63	12	**28**	55	0.95	**0.000**
Santalales	2	2	1	1	0.00	0.990
Sapindales	21	5	**10**	19	0.94	**0.000**
Saxifragales	4	2	4	3	0.00	0.990
Selaginellales	3	2	2	2	1.00	**0.001**
Solanales	42	8	**12**	37	0.89	**0.000**
Vitales	2	1	2	2	0.00	0.990
Zingiberales	15	3	14	13	0.24	0.580

In the following description of how utilization of these orders is distributed in the family Nymphalidae, we make use of the informal clade names “satyrines,” “heliconiines,” and “nymphalines,” which unite related subfamilies into three large nymphalid clades, according to Wahlberg et al. (2009).

*Rosales*: This is not the most frequently used order in terms of number of genera feeding on it (Table 2), but it is the only serious candidate to being the ancestral nymphalid host order (at least if the ancestor used a single order for its hosts). It is used by the basal subfamily Libytheinae as well as by some Danainae, by Calinaginae and other basal “satyrines,” by many “heliconiines” and very prominently by “nymphalines.” In the simplified tribe-level tree it shows up as being the ancestral host order (Fig. 1). With hosts reconstructed on the complete genus-level tree using an unordered matrix, it is the only order reconstructed as the ancestral host, although the state of the character is still equivocal (results of these analyses are not shown, but see Appendices S2 and S3 for the data files). Note, however, that this reconstruction of each order as a separate character permits zeroes for all plant orders, ignoring the fact that something must have been used as host. Using the “costly gains” step matrix, Rosales is unequivocally reconstructed as the ancestral host, and there is no other candidate.

*Malpighiales*: This host order is actually used even more frequently than Rosales (Table 2), but in a phylogenetically somewhat more apical manner (Fig. 1). It is not used by the basal Libytheinae or Danainae, but by Charaxinae among the “satyrines,” by some “nymphalines” (in particular Biblidinae), and very prominently by “heliconiines.” With host orders reconstructed on the complete phylogeny using an unordered matrix, use of Malpighiales as host is the ancestral state in Biblidinae, Charaxinae, and in the entire heliconiine clade. With the “costly gains” matrix it is reconstructed as a possible ancestral host for all Nymphalidae except the basal clades Libytheinae and Danainae, and with the “very costly gains” matrix (gains weighted five times more difficult than losses) feeding on Malpighiales becomes the ancestral state for the whole family. It is worth noting that besides Rosales and Malpighiales, only one other order is reconstructed as an ancestral host (i.e., part of a range of hosts) even with the extreme weighting of gains in the “very costly gains” matrix, namely Gentianales (with Solanales being an additional equivocal candidate).

*Gentianales*: Use of this host is characteristic for Danainae, where it seems to be the ancestral host order (Willmott and Freitas 2006), but it is also frequently used elsewhere in the Nymphalidae. Within the danaine tribe Ithomiini there is a switch from Gentianales to *Solanales* as the characteristic host order (Fig. 1; Willmott and Freitas 2006). It is used also by scattered genera elsewhere in the family.

*Arecales* (palms) is the most important host order for many basal “satyrine” clades. This monecotyledon order is very rarely used in other parts of Nymphalidae. Later, there were one or several switches to *Poales* (grasses) as the characteristic host order in the extensive radiation of satyrine butterflies (Fig. 1; Pena and Wahlberg 2008; Pena et al. 2011). Thus, although Poales is almost exclusively used in satyrines, it is actually the most frequently used host order in terms of butterfly genera (Table 2).

*Lamiales*: There are scattered uses of this order in many parts of the nymphalid tree, but it is most characteristic of Melitaeini and related genera in the Nymphalinae, following a switch from Rosales as main host order (Fig. 1; Nylin and Wahlberg 2008).

#### Butterfly host range

Figure 2 shows the phylogenetic position of polyphagous species (feeding on at least three orders) mapped onto the simplified tribal-level phylogeny (data in Table 1). Note that a single polyphagous species is enough for a tribe to be shown as polyphagous (e.g., a single species in Satyrini, containing nearly 25% of all nymphalid species, was found to feed on three orders); nevertheless, the character has a very apical distribution in the phylogeny. The seeming exception of the Limenitidinae is another artifact of the simplified reconstruction: there are only 10 species in Neptini, four species in Adoliadini, and three species in Limenitidini known to feed on three orders or more, out of a total of several hundred species.

Our database of the Nymphalidae contains 551 butterfly taxa, typically at the genus level (see Materials and Methods). Host plant data is wholly lacking for 133 of these taxa. Of the remaining 418 taxa, 71 contain at least one species feeding on at least two host orders, 30 (of these 71) contain at least one species feeding on at least three host orders, and in the remaining 347 taxa (83%) there are no species at all known to be polyphagous (using these criteria). Although such species may well remain to be found, this clearly demonstrates that most nymphalids are relatively specialized in their host use.

Another way to show this is at the species level: there are about 6000 described species in the family (Maddison and Schulz 2007), but we found only 249 species with records of feeding on more than one plant order, and out of these only 79 have been indicated to feed on at least three orders (Table 1). For our purposes, furthermore, only the latter type of species can be seen as candidates for being “truly” polyphagous, that is opportunistic generalists, because it is a common pattern in Nymphalidae that a couple of ancestral clade-specific host orders are used in parallel—a pattern rather more indicative of conservative host use than opportunism.

Even among the 79 species feeding on at least three orders, few fit the “generalist” description well, but there are a few obvious candidates in the genera *Hypolimnas*, *Vanessa*, *Polygonia*, and *Charaxes*. These will be examined in more detail in the case studies later.

### HOST RANGE VERSUS HOST ORDERS

#### General patterns

Although the complete phylogeny is too large to be shown here (see however Fig. S1), it should be noted that even a superficial view of the polyphagy characters (feeding on) “2 orders” and in particular “3 orders” optimized onto the phylogeny even more clearly reveals the apical nature of the state of polyphagy than the tribal-level phylogeny shown in Figure 2 (see Table 3 for a summary). For the former character, there are a number of clades where “polyphagy” is unequivocally reconstructed as being shared by one or more related taxa due to common ancestry, but none of these shared states go deeper than three nodes into the phylogeny. For the latter character, there are only two such clades, and they go only a single node into the phylogeny. This is despite the fact that a single polyphagous species is enough to qualify a higher taxon for this state, and despite the fact that the actual host orders involved could be any of the many used by nymphalids and could be different in the related taxa, strongly exaggerating the chances for the state to be optimized as ancestral (explaining why polyphagy seemingly goes deeper into the simplified phylogeny; Fig. 2).

**Table 3:** Cases in which a state is shared among related genus-level taxa of nymphalid butterflies and reconstructed as having been inherited from a common ancestor, highlighting the clades where the state is reconstructed as having been present for the highest number of successive nodes in the phylogeny counting from the tips down (“deepest node”)

Character	No. of cases	Deepest node	Age (Mya)	Clade(s)
“2 orders”	12	3	17	*Argynnis, Boloria* and relatives,
			20	*Euryphura* and related genera
“3 orders”	2	1	32	*Acrea + Actinote*
			20	*Faunus* + *Taenaris*
Rosales	5	12	72	“nymphalines”
Malpighiales	3	9	68	“heliconiines”
Arecales	1	8	49	Brassolini + Morphini and relatives
Lamiales	3	11	50	Melitaeini

This can be contrasted with the situation when individual host orders are optimized onto the phylogeny. A relevant comparison would be with host taxa used about as rarely as the states of polyphagy, treating the characters as unordered, that is with equal weighting of gains and losses. Selecting host orders with the most similar lower and higher frequency, respectively, the character “2 orders” (71 taxa) can be contrasted with Rosales (63 taxa) and Malpighiales (78 taxa), and the character “3 orders” (30 taxa) can be contrasted with Arecales (24 taxa) and Lamiales (41 taxa). As noted earlier, all of these host orders are principal hosts for the family. Use of Rosales is reconstructed as independently shared among related taxa in only five clades, but in one of the major clades of nymphalids the shared state goes unequivocally very deep into the genus-level phylogeny (Table 3). Regarding use of Malpighiales, there are only three cases, but in each case these are major clades where the shared state goes deep into the phylogeny (“heliconiines,” Biblidinae, Charaxinae). Shared use of Arecales or Lamiales among relatives is also rare, but goes much deeper into the phylogeny than the two states measuring polyphagy (Table 3).

Similarly, dating the phylogeny by constraining node ages as described under Methods suggests that the deepest shared nodes are considerably older in the case of individual host plant orders than for the two “polyphagy” traits (Table 3).

#### Phylogenetic signal

The character (feeding on) “2 orders” showed a significant phylogenetic signal, using either the parsimony randomization test or the λ estimate, whereas the character “3 orders” did not (Table 2). We suggest that this is in line with the notion that host orders are most often inherited from an ancestor rather than being opportunistically colonized. Note in the database (Appendix S1), for instance, that in the tribe Limenitidini there are several genera with species feeding on two host orders, but these are typically pairwise combinations of a few host orders characteristic of the tribe, in particular Rosales, Malpighiales, Dipsacales, and Gentianales. In the Junoniini, use of the principal host Lamiales is combined with one other host order in several genera, and the same is true for Malpighiales in the Argynnini. Finally, Arecales, Poales, and Zingiberales are pairwise combined in many satyrine genera. These patterns suggest that a few clade-specific host plant taxa can sometimes be combined over long evolutionary times to create a phylogenetic signal for the character “2 orders,” but that using three orders (or more) is a more unstable state that tends to degenerate into a more specialized state.

Turning to the host orders themselves, many of these showed a significant phylogenetic signal, indicating a conserved pattern of host use among genera (Table 2). Use of Arecales, Fabales, Gentianales, Lamiales, Malpighiales, Poales, Rosales, Sapindales, and Solanales showed a significant signal regardless of method. This corresponds closely to the principal nymphalid hosts mentioned earlier, with the exception of Fabales and Sapindales. Fabales (or an ancestor of the order) is the likely ancestral host taxon for the butterflies as a whole (Janz and Nylin 1998), and although it is less important in the Nymphalidae than in other families it is still prominently used in for instance the tribes Neptini and Charaxini, where it is shared among genera. Use of Sapindales is a bit scattered, but it is shared among genera in, for example, the Adoliadini, Epiphilini, and Callicorini, causing the significant phylogenetic signal.

#### Retention index

The retention index gives some indication of the homoplasy of a character, but it is a somewhat blunt instrument in that it is highly dependent on how widespread among taxa the character is. Thus, we found the highest number (1, the maximum) for Commelinales, a host used by only two related taxa, and high numbers also for Liliales and Selaginellales, each used by only three taxa (Table 4). More interestingly, however, we found indices at or above 0.48 (the total value for all characters in the matrix) for the following additional host orders, all used by many taxa: Arecales, Gentianales, Lamiales, Malpighiales, Poales, Rosales, Sapindales, and Solanales. This again highlights the same principal nymphalid hosts.

**Table 4:** Retention index (parsimony) and apicality (ML) for polyphagy and host order utilization in nymphalid butterflies. Italics show retention indices at or above the total for all characters (0.48) and delta estimations below 3

Character	No. of butterfly taxa	Retention index	Delta estimation
2 orders	71	0.16	4.1
3 orders	30	0.10	10
Arecales	24	*0.48*	*1.5*
Aspergales	4	0.33	3.2
Asterales	12	0.09	10
Boraginaceae	3	0.00	10
Brassicales	2	0.00	10
Caryophyllales	9	0.13	5.0
Celastrales	4	0.00	10
Commelinales	2	*1.00*	*0.2*
Cucurbitales	2	0.00	10
Dipsacales	6	0.00	10
Ericales	16	0.07	10
Fabales	12	0.27	*1.9*
Fagales	13	0.08	10
Gentianales	15	*0.71*	*0.2*
Lamiales	41	*0.55*	*2.7*
Laurales	16	0.07	8.2
Liliales	3	*0.50*	*2.3*
Magnoliales	3	0.00	*2.5*
Malpighiales	78	*0.67*	*0.7*
Malvales	13	0.17	4.2
Myrtales	14	0.08	10
Oxalidales	3	0.00	10
Pandanales	4	0.33	4.2
Piperales	4	0.00	10
Poales	109	*0.87*	*0.5*
Ranunculales	4	0.00	4.4
Rosales	63	*0.56*	*0.0*
Santalales	2	0.00	10
Sapindales	21	*0.55*	*1.8*
Saxifragales	4	0.00	10
Selaginellales	3	*0.50*	*0.4*
Solanales	42	*0.73*	*1.8*
Vitales	2	0.00	10
Zingiberales	15	0.07	10

In contrast, the characters (feeding on) “2 orders” and “3 orders” had retention indices below 0.2, suggesting that host range (polyphagy) is less conserved than host order.

#### Apicality

The host range character “3 orders” (polyphagy) showed a high delta estimate, indicating apicality (i.e., the character is not shared among several related taxa; Table 4). Similarly to results for “phylogenetic signal,” the lower delta estimate for the character “2 orders” indicates that this trait is more often shared by relatives.

An apical distribution was found also for several host orders, but only if they are uncommonly used. All host orders with high (maximum) delta estimates are fed upon by less than 16 taxa, in most cases much less. In contrast, 14 host orders showed very low delta estimates (phylogenetically conserved patterns, arbitrarily below 3) and half of these are used by more than 16 taxa (the remaining taxa included the rarely used Comellinales, Selaginellales, and Liliales; cf. last section). This comparison is somewhat circular in that hosts used by many taxa would tend to show a less apical distribution for purely statistical reasons (Stireman 2005). More interestingly, the host range character “3 orders” is shared by as many as 30 taxa, and still showed a high delta estimate (Table 4).

When branch lengths were arbitrarily made ultrametric the delta estimates tended to be higher overall, and was at the maximum of 10 for the character “2 orders,” but otherwise the results were qualitatively similar (not shown).

#### Transitions

The exact transition rates between monophagy (feeding on a single host order) and polyphagy (feeding on at least two or at least three host orders, respectively) was dependent on the resolution of a few ambiguous nodes in the phylogeny. We first maximized transitions to monophagy, and then minimized them (the latter numbers in brackets).

In the family Nymphalidae we found 26 (28) transitions to a more polyphagous state using the character (feeding on) “3 orders” but only 2 (0) transitions from polyphagy to monophagy. This difference is highly significant (*χ*^2^ = 20.6, df = 1, *P* < 0.001 or *χ*^2^ = 28.0, df = 1, *P* < 0.001 minimizing transitions to monophagy).

Using instead the character “2 orders” there were 44 (56) transitions to polyphagy and 17 (5) transitions to monophagy. The difference is significant when transitions to monophagy are maximized (*χ*^2^ = 12.0, df = 1, *P* < 0.001) as well as when they are minimized (*χ*^2^ = 42.6, df = 1, *P* < 0.001).

#### Comparisons to Dendroctonus

The bark beetle genus *Dendroctonus*, in which specialization has instead been suggested to be the apical state, (Kelley and Farrell 1998) provides an interesting comparison, albeit at a completely different phylogenetic level. In the genus, there are 12 “polyphagous” species and a number of species specializing on different species of *Pinus* or *Picea* trees (Table 5).

**Table 5:** Phylogenetic signal in polyphagy and host species utilization in *Dendroctonus* bark beetles, according to parsimony (randomization test) and ML. The “no. of bars” (in the histogram resulting from the randomization test) gives an indication of the variation in number of steps over 1000 random trees. When no. of steps in characters fall below percentile boundaries they are significant at *P* < 0.05 (marked in bold). ML lambda estimates significantly different from 0 with a *P*-value of <0.05 are marked in bold

		Parsimony			ML	
Character	No. of butterfly taxa	No. of bars	Steps in char.	percentile boundary (0.05)	Lambda estimate	*P*-value
Polyphagy	12	4	6	3	0.00	0.990
*Pinus monticola*	2	2	2	1	0.00	0.990
*Pinus strobus*	4	4	4	2	0.00	0.990
*Pinus lambertiana*	2	2	2	1	0.00	0.990
*Pinus ayacahuite*	3	3	3	1	0.00	0.990
*Pinus edulis*	2	2	2	1	0.00	0.990
*Pinus leiophylla*	5	4	5	2	0.00	0.990
*Pinus palustris*	2	2	2	1	0.00	0.990
*Pinus taeda*	2	2	2	1	0.00	0.990
*Pinus echinata*	3	3	3	1	0.00	0.990
*Pinus rigida*	3	3	3	1	0.00	0.990
*Pinus ponderosa*	7	6	4	3	0.80	0.120
*Pinus jeffreyi*	2	2	2	1	0.00	0.990
*Pinus engelmannii*	3	3	3	1	0.00	0.990
*Pinus montezumae*	4	4	4	2	0.00	0.990
*Pinus hartegii*	3	3	3	1	0.00	0.990
*Pinus pseudostrobus*	4	3	3	2	0.95	0.870
*Pinus teocote*	2	2	2	1	0.00	0.990
*Pinus lawsonii*	2	2	2	1	0.00	0.990
*Pinus coulteri*	3	3	3	1	0.00	0.990
*Pinus contorta*	3	3	3	1	0.00	0.990
*Pinus virginiana*	2	2	2	1	0.00	0.990
*Pinus oocarpa*	4	3	4	2	0.00	0.990
*Pinus tenufolia*	4	3	4	2	0.00	0.990
*Pinus rudis*	4	4	3	2	1.00	0.200
*Picea sitchensis*	3	2	2	2	0.97	0.150
*Picea engelmanii*	2	2	2	1	0.00	0.990
*Picea glauca*	3	2	2	2	0.97	0.150
*Picea mariana*	2	2	2	1	0.00	0.990
*Picea rubens*	2	2	2	1	0.00	0.990

Using the same methods as for Nymphalidae, we found no significant phylogenetic signal in this data matrix, probably reflecting its small size as well as the dynamic patterns of host use (Table 5). Similarly, the retention indices were zero for both the trait “polyphagy” and for most host species (Table 6). Five host species showed higher retention indices (above the total for all characters, which was 0.12), indicating less homoplasy, and the same species were the only ones with delta estimates of apicality that were not at the maximum for the analysis, that is which showed some degree of phylogenetic conservatism.

**Table 6:** Retention index (parsimony) and apicality (ML) for polyphagy and host order utilization in *Dendroctonus* bark beetles. Italics show retention indices above the total for all characters (0.12) and delta estimations below 5

Character	No. of beetle taxa	Retention index	Delta estimation
Host range	12	0	10
*Pinus monticola*	2	0	10
*Pinus strobus*	4	0	10
*Pinus lambertiana*	2	0	10
*Pinus ayacahuite*	3	0	10
*Pinus edulis*	2	0	10
*Pinus leiophylla*	5	0	10
*Pinus palustris*	2	0	10
*Pinus taeda*	2	0	10
*Pinus echinata*	3	0	10
*Pinus rigida*	3	0	10
*Pinus ponderosa*	7	*0.5*	*0.84*
*Pinus jeffreyi*	2	0	10
*Pinus engelmannii*	3	0	10
*Pinus montezumae*	4	0	10
*Pinus hartegii*	3	0	10
*Pinus pseudostrobus*	4	*0.33*	*4.19*
*Pinus teocote*	2	0	10
*Pinus lawsonii*	2	0	10
*Pinus coulteri*	3	0	10
*Pinus contorta*	3	0	10
*Pinus virginiana*	2	0	10
*Pinus oocarpa*	4	0	10
*Pinus tenufolia*	4	0	10
*Pinus rudis*	4	*0.33*	*2.05*
*Picea sitchensis*	3	*0.5*	*2.31*
*Picea engelmanii*	2	0	10
*Picea glauca*	3	*0.5*	*2.31*
*Picea mariana*	2	0	10
*Picea rubens*	2	0	10

Regarding transitions, in contrast to the butterflies there were no transitions from monophagy to polyphagy, but six transitions to monophagy (df = 1, *P* < 0.05 using Fisher's exact test). The pattern of transitions in the beetles is furthermore significantly different from the one in the butterflies using Fisher's exact test, regardless of polyphagy character (“2 orders” or “3 orders”) and regardless of how equivocal nodes were resolved (df = 1, *P* < 0.001 for “3 orders” and for “2 orders” when transitions to monophagy are minimized; *P* < 0.01 for “2 orders” when they are maximized).

#### Butterfly case studies

Four of the taxa in our database contain several polyphagous species, and also what seems to be the most polyphagous single species. These taxa are *Nymphalis*/*Polygonia*, *Vanessa*, *Hypolimnas*, and *Charaxes*.

The *Nymphalis-Polygonia* group of species (part of tribe Nymphalini in the Nymphalinae) has been extensively studied elsewhere (Janz et al. 2001; Weingartner et al. 2006). We have demonstrated that the ancestor of this clade most likely was a specialist on Rosales (more specifically families belonging to the “urticaelean rosids”: Urticaceae, Ulmaceae, Cannabaceae). Host use was then widened to include also a number of other plant families shared by several butterfly species (Rosaceae in the Rosales, Betulaceae in the Fagales, Salicaceae in the Malpighiales, Grossulariaceae in the Saxifragales, Ericaceae in the Ericales). The set of hosts includes all plant growth forms: herbs, vines, bushes, and trees. Many species have since re-specialized on a subset of these shared taxa, or on odd plants that may have been colonized during the more generalist phase of this oscillation in host range. However, species such as *Polygonia c-album* and *Polygonia faunus* remain polyphagous on several of the “typical” host orders, albeit with variation among local populations in the precise range used (Nylin et al. 2009; Kodandaramaiah et al. 2012).

*Conclusion*: Not even the most polyphagous species in this clade are well described as true generalists. Rather they feed on a range of clade-specific orders shared with other species in the clade. There is some evidence that diversification in the *Nymphalis-Polygonia* group was aided by host range fluctuations, and there are more species in the clade than in the specialized sister clade (Table 7; data from Weingartner et al. 2006).

**Table 7:** Number of species in clades of Nymphalidae containing the larger fraction of, or the most strongly polyphagous species, as compared to the more specialized probable sister clade

Clade contrast	No. of species in most polyphagous clade	No. of species in sister clade
*Nymphalis-Polygonia* vs. *Aglais s. l*.	∼20	∼5
*Vanessa* vs. *Hypanartia*	19–22	14
*Hypolimnas* vs. *Precis*	25	17
*Charaxes* vs. *Polyura + Euxanthe*	200+	30
*Charaxes (wide) + Palla* vs. *Prothoini*	234+	5

The *Vanessa* group (another part of the tribe Nymphalini) may have had a similar evolutionary history (Figs. 3, 4; J. Slove, T. Eriksson, S. Nylin, N. Janz, in prep.). Again the ancestor was in all probability a specialist on the “urticalean rosids” section of Rosales (in this case more specifically the family Urticaceae) and again the host plant range was widened to a shared set of families, but a completely different set from the previous clade (in this case all hosts are herbs; besides Urticaceae: Asteraceae in the Asterales and Malvaceae in the Malvales). *Vanessa cardui* is one of the most polyphagous butterfly species, feeding on a wide range of additional families and orders (Fabales, Vitales, Cucurbitales, Caryophyllales, Lamilales, Solanales, the unplaced Boraginaceae, perhaps more) besides these shared ones. Note from Figures 3 and 4, the apical distribution of polyphagy and of the use of the plant orders Asterales and Malvales, reconstructed as having been colonized independently several times by specialists on Rosales. Similarly to the case for *Polygonia* (Janz et al. 2001; Weingartner et al. 2006), however, we suggest that it is more likely that adaptations to use of Asterales and Malvales (and hence polyphagy as defined here) evolved early in the *Vanessa* clade, but that later respecialization by most species has obscured this pattern to the extent that it cannot now be easily reconstructed.

*Conclusion*: The cosmopolitan *V. cardui* can be described as a generalist, perhaps indicative of a recent host expansion, but even in this case the preferred hosts are the shared clade-specific ones. There are 19 species of *Vanessa* listed on the Tree of Life site (Maddison and Schulz 2007), and 22 are listed by Wahlberg and Rubinoff (2011). The most likely sister genus, *Hypanartia* (Wahlberg and Rubinoff 2011), is specialized on urticalean rosids and contains fewer species (Table 7; Nylin and Wahlberg 2008). There is however considerable variation in host range also within *Vanessa*, and the phylogenetic resolution and even the placement of species in the genera *Vanessa*, *Hypanartia*, and *Antanartia* is still in some flux (Wahlberg and Rubinoff 2011), making it difficult at this stage to deduce whether polyphagy has aided diversification.

*Hypolimnas* (part of the tribe Junoniini in the Nymphalinae) is a genus containing several polyphagous species. There is no published phylogeny for the genus, but the main patterns of host plant evolution can still be reconstructed. The urticalean rosids was the ancestral host for Nymphalinae as a whole, with a later shift to the order Lamiales via a polyphagous stage (Nylin and Wahlberg 2008). *Hypolimnas* belongs to the part of Nymphalinae that have shifted to Lamiales as principal host, and all of the most closely related genera such as *Precis*, *Yoma*, and *Junonia* feed primarily on this host order. It has been suggested that Urticaceae is the likely ancestral host family for *Hypolimnas* (Vanewright et al. 1977), but, if so, this is a re-colonization at the base of the genus. Within *Hypolimnas*, some species specialize on Lamiales hosts, some on Urticaceae, and some use both host taxa. Other species such as *Hypolimnas bolina*, *Hypolimnas missipus*, and *Hypolimnas alimena* are more polyphagous, feeding also on other shared taxa (Malvales; Caryophyllales; Solanales) as well as some odd taxa in the case of *H. bolina* (Gentianales, perhaps Fabales, Commelinales).

*Conclusion*: Similarly to *V. cardui*, even the polyphagous species in *Hypolimnas* seem to prefer the shared clade-specific orders, but the widespread *H. bolina* and *H. missipus* could perhaps be described as generalists. There are more species in the genus *Hypolimnas* currently listed at the Tree of Life site (Maddison and Schulz 2007) than in the more specialized sister genus *Precis* (Table 7). It would be very interesting to reconstruct the host plant utilization and diversification in this clade in more detail when phylogenetic information becomes available. If polyphagy evolved only recently in the clade (Vanewright et al. 1977 suggest that *H. bolina* and *H. missipus* may be sister species), there may however not yet have been time for plasticity to promote much diversification.

*Charaxes* (part of tribe Charaxini in the Charaxinae) is a very diverse genus (over 200 species listed at the Tree of Life site), with most species being African, although the genus extends into Asia. Species limitations are often problematic in the genus, and many species may be paraphyletic (Müller et al. 2010). There are recent phylogenetic analyses from Aduse-Poku et al. (2009) and (for the Asian taxa) Müller et al. (2010). The former study included over half of the *Charaxes* species, including 83 African, as well as representatives of the genera *Polyura* and *Euxanthe* (which were found to be most likely nested within *Charaxes sensu latu*). In our database there are 33 *Charaxes* species feeding on at least two orders and 19 of these feed on at least three orders. In *Polyura*, seven species (out of 24) feed on at least two orders, and two of those on three orders or more. In *Euxanthe*, two species (out of six) feed on two orders. Moreover, there are records from 19 different host orders in total for the genus *Charaxes* (including *Polyura* and *Euxanthe*). There is however some order even to these seemingly chaotic butterfly–plant relationships. In *Polyura* and *Euxanthe*, the principal hosts are Fabales (both genera), Sapindales (primarily *Euxanthe*), and Rosales (primarily *Polyura*). These orders are very commonly used by *Charaxes* as well; Fabales indeed by almost all polyphagous species and by many specialists. The most common additional host orders for *Charaxes* are Poales, Malpighiales, Myrtales, and Celastrales, and subsets of these seven orders are shared by many polyphagous species.

*Conclusion*: The most polyphagous currently recognized species are *Charaxes castor* and *Charaxes epijasius*. At least these two species (which are relatively closely related; Aduse-Pooku et al. 2009) could be described as generalists, but it is noteworthy that most of their host orders are actually used also by other *Charaxes* species. There are over 200 species in the genus *Charaxes* and an additional 30 if *Polyura* and *Euxanthe* are included. Because polyphagy is very widespread in the clade, this diversity supports a link between host niche width and diversification. However, the number of species cannot be straightforwardly contrasted with a sister clade because of the phylogenetic uncertainty. If the sister clade to *Charaxes* is *Polyura + Euxanthe* (as was previously believed), this provides a contrast in some support of our hypothesis, because polyphagy is less extreme in this more species-poor clade (Table 7). In the analysis of Aduse-Poku et al. (2009), the genus *Palla* emerged as a possible sister clade of the wider *Charaxes* clade, and thus provides another possible contrast, but out of the only four species in the genus one feeds on three orders and two on two orders, meaning that the genus is not clearly less polyphagous than *Charaxes*. Finally, the entire clade *Charaxes* + *Polyura* + *Euxanthe* + *Palla* can be contrasted with its possible sister clade *Prothoe* + *Agatasa* (Wahlberg et al. 2009), a species-poor clade (the Prothoini) with no known polyphagous species (Table 7).

## Discussion

Our analyses reveal a surprisingly clear and simple pattern of larval host plant utilization in the Nymphalidae at the level of plant orders. The “urticalean rosids” section of Rosales (hackberry trees, elms, nettles, and their relatives) is robustly reconstructed as the ancestral host plant association in Nymphalidae. This is in contrast to the other butterfly families, where another group of rosids—the legumes (Fabales)—apparently was the ancestral host (Janz and Nylin 1998), and suggests that the colonization of urticalean plants was a key event that perhaps helped pave the way for the evolutionary success of nymphalid butterflies.

A few additional later colonizations also contributed to shaping a very large part of the butterfly–plant associations observed in the family today. The order Gentianales was colonized by an ancestor of the subfamily Danainae at some point between 94 and 52 Mya, followed by a switch to another Euasterid clade, Solanales, in an ancestor of the species-rich tribe Ithomiini about 46–37 Mya (Willmott and Freitas 2006; datings based on Wahlberg et al. 2009). In the “satyrine” section of nymphalids, a shift to monocotyledon hosts (palms in Arecales), and later to grasses, has apparently been of tremendous importance for its diversification (Pena and Wahlberg 2008; Pena et al. 2011). Within the subfamily Nymphalinae, the order Lamiales was colonized about 50 Mya, and later the Asterales was colonized by an early member of the tribe Melitaeini, and there are more species in the taxa with the wider host use (Nylin and Wahlberg 2008).

Finally there is the host order Malphigiales (Passifloraceae, Violaceae, Euphorbiaceae, and relatives), showing a more complex phylogenetic pattern. Hosts in this order are used by butterflies in several sections of the family, in particular in the subfamilies Heliconiinae, Biblidinae, and Charaxinae but also elsewhere, and it is not clear whether there have been several truly independent colonizations or whether early nymphalids may have fed on both urticalean hosts and plants in Malphigiales (both groups belong to the Eurosid I clade sensu Stevens 2001, as indeed do the legumes). In any case, it is intriguing that despite the large size of the family and the roughly 100 million years time span involved (Wahlberg et al. 2009), only a handful of plant orders can be implicated as having played an important role in its evolution. This is in line with the suggestion, put forward by Ehrlich and Raven already in 1964, that butterflies and many other phytophagous insects have been historically constrained by the chemical characteristics of their host plants. These authors also suggested that shifts to novel plant taxa—new “adaptive zones”—may have spurred diversification of butterflies, a pattern that has received later support (Willmott and Freitas 2006; Wheat et al. 2007; Pena and Wahlberg 2008; Fordyce 2010; Pena et al. 2011). However, we have proposed that what long after the event looks like simple host shifts may actually have been polyphagous stages during which novel hosts are more likely to be colonized (Janz and Nylin 2008; Nylin and Wahlberg 2008).

Against this background, we studied the phylogenetic distribution of polyphagous species among nymphalid butterflies, testing our prediction that plastic host use is a transient state in evolution. We showed that there are few polyphagous species in the family, when they are defined as feeding on more than one host order, and in particular if the criterion is at least three orders represented among the hosts. The latter character also showed no significant phylogenetic signal, in contrast to the use of the actual host orders involved, which was often highly constrained by phylogeny. This was especially true for the principal hosts mentioned earlier, and the same host orders also showed high retention indices (low degrees of homoplasy) and low estimates of “apicality” (i.e., the character was instead shared among relatives), particularly compared to the characters denoting polyphagy.

The reconstructed transitions in host range were also in line with a transient nature of polyphagy, although this may not be evident at first sight: there were many more transitions from monophagy to polyphagy than the reverse among nymphalid butterflies, at least when the more conclusive “3 orders” criterion was used, again indicating apical positions of wide host ranges in the phylogeny. Importantly, we suggest that it is very unlikely that this reflects unidirectional evolution or a recent burst in the evolution of polyphagous species. Rather, the pattern is likely to be due to oscillations in host range, where recently evolved cases of plastic host use still remain in evidence, whereas the widespread reversals to monophagy in the more distant past now leave no trace other than (sometimes) host shifts. In other cases, respecialization would instead have been onto the ancestral host order, as it is likely to often remain the superior host, and this would erase all traces of past polyphagy, leading to the seemingly simple patterns of host use that we can reconstruct today.

Overall, there seems to be a trend toward specialization in phytophagous insects, but in some taxa the opposite trend is observed (Nosil 2002). The family Nymphalidae is one such exception, if the observed patterns are taken at face value. However, Stireman (2005) cautioned that artefacts due to reconstruction methods can severely bias such results. In fact, Stireman reported a very similar pattern of apical polyphagy from parasitoid tachinid flies, but was reluctant to conclude that there is an actual evolutionary trend toward generalization, or a higher extinction probability for generalists—counter to the prevailing paradigm in the field. Instead, he suggested various other possibilities, including a model where extinction rates are lower for generalists, and where generalists are more prone to diversify, but in so doing produce specialized descendants rather than additional generalist lineages. He showed that this can produce phylogenies where generalists are reconstructed as apical, even though in actuality the generalist stage persists over long evolutionary time spans.

Stireman's model is in fact similar to our view of evolution in nymphalid butterflies (Janz and Nylin 2008), although we would add that by assuming oscillations in host range the apparent conflict between an overall trend toward specialization and apical polyphagy in the same taxon is resolved. Also, we suggest the strong possibility that the “persistent” generalist stage may not always be actualized over evolutionary time, but rather reflect a potential for repeatedly recolonizing a set of shared clade-specific hosts used by ancestors (Nylin and Wahlberg 2008; Agosta et al. 2010), a case of “recurrence homoplasy” due to shared ancestry, in the terminology of West-Eberhard (2003).

The comparison with the bark beetle genus *Dendroctonus* is interesting, because it shows a taxon where (according to our analysis) most transitions are instead from polyphagy to monophagy, in line with the interpretation of the original authors (Kelley and Farrell 1998) but in sharp contrast to the reanalysis of Nosil (2002). Nosil's result may be an artefact (Stireman 2005), and we prefer the interpretation of increased specialization over time, which is more intuitively true given the completely apical distribution of monophagy in the phylogeny. We suggest, however, that this pattern is equally consistent with the oscillation hypothesis; it simply catches evolution in a later stage of an oscillation, where species are again specializing.

Indeed, as shown by the more detailed case studies of butterfly clades containing polyphagous species that we have presented, this is simply a matter of scale. In each of these cases, there is evidence that host use in the clade was initially restricted to one or at most a few host orders. More polyphagous species evolved more recently, but most of these species are not true opportunistic generalists, but rather make use of a limited number of shared plant taxa. A few species are exceptions, including also several other odd taxa among their hosts, and these species are the ones that we suggest are most likely to become widespread because they can make use of a diverse range of locally available hosts (as is indeed the case for *V. cardui, H. bolina, H. missipus*, and *C. castor*) and later give rise to locally adapted specialist species, in accordance with the oscillation hypothesis (Janz and Nylin 2008). The evidence that such diversification has already happened in these clades is not very strong, but there are consistently more species in the genera containing the polyphagous species than in the sister clades. At the very least, this suggests that plasticity in host plant utilization does not oppose speciation by providing an alternative to genetic differentiation, but rather supports the general conclusions from earlier studies using the total diversity of host use in clades rather than the actual host ranges of species to indicate plasticity (Janz et al. 2006; Nylin and Wahlberg 2008). It would be of great interest to similarly analyze the interesting patterns of fluctuations in host range in other butterflies, such as the Papilionidae (Scriber 2010), and in other phytophagous insects, such as pollen-feeding bees (Sedivy et al. 2008), for impact on diversification.

## Conclusions

The phylogenetic distribution of the polyphagous state among nymphalid butterflies is strongly indicative of a state which is transient and prone to quickly—in evolutionary terms—disappear due to selection in favor of specialization on a more limited set of related, and hence chemically similar, plants. This means that the family presents numerous evolutionary “experiments” with which the oscillation hypothesis as well as West-Eberhard's more inclusive theory of plasticity as a driver of diversification can be critically tested, as species-level phylogenies and detailed host information becomes available.

## Supporting Information

Appendix S1 The database of polyphagous species in the family Nymphalidae, in Excel format.

Appendix S2 The character matrix for the two traits scoring polyphagy and use of host plant orders, for each higher butterfly taxon in the family Nymphalidae, in Excel format.

Appendix S3 Nexus file containing the character matrix and the phylogenetic tree of the family Nymphalidae used in the analyses.

Figure S1 Figure (in pdf format) showing the character “use of three host plant orders” optimized onto the phylogeny of the family Nymphalidae, using parsimony and treating the character as unordered.
