# Supplementary figures and images for: HOST PLANT UTILIZATION, HOST RANGE OSCILLATIONS AND DIVERSIFICATION IN NYMPHALID BUTTERFLIES: A PHYLOGENETIC INVESTIGATION
Source: Evolution. 2013 Aug 29;68(1):105–24. doi: 10.1111/evo.12227 (PMC3912913; doi:10.1111/evo.12227)

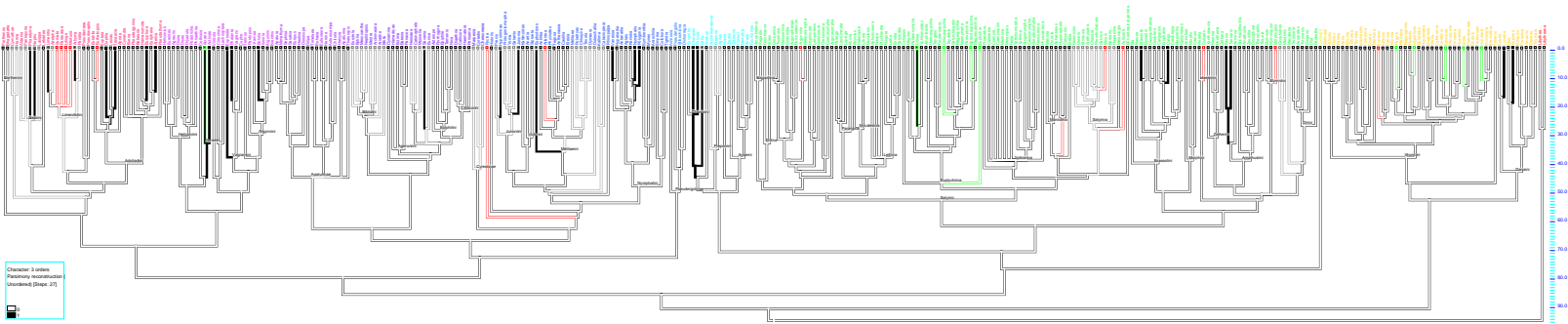

Supplement: Figure S1 — Figure (in pdf format) showing the character “use of three host plant orders” optimized onto the phylogeny of the family Nymphalidae, using parsimony and treating the character as unordered. [file evo0068-0105-sd4.pdf]
